# Utilization of adolescent friendly health services and its associated factors among higher secondary students in mid-western Himalayan mountainous district of Nepal

**DOI:** 10.1371/journal.pgph.0001616

**Published:** 2023-03-17

**Authors:** Mahesh Sharma, Bijay Khatri, Archana Amatya, Narayan Subedi, Dipak Prasad Upadhyaya, Bhim Prasad Sapkota, Parvati Bista

**Affiliations:** 1 United Nations Development Programme Nepal, Pulchowk, Lalitpur, Nepal; 2 Ministry of Health and Population, Kathmandu, Nepal; 3 Academic & Research Department, Hospital for Children, Eye, ENT, & Rehabilitation Services (CHEERS), Madhyapur Thimi, Bhaktapur, Nepal; 4 Save The Children, Health and Nutrition, Nepal; 5 Central Department of Public Health, Institute of Medicine (IoM), Tribhuvan University, Kathmandu, Nepal; 6 Department of Population and Quantitative Health Sciences, Case Western Reserve University, Cleveland, Ohio, United States of America; 7 School of Public Health, B.P. Koirala Institute of Health Science, Dharan, Sunsari, Nepal; The University of Texas Health Science Center at Houston School of Public Health - San Antonio Campus, UNITED STATES

## Abstract

Adolescent friendly health services (AFHS) are designed to make health services accommodate the unique needs of adolescents. AFHS are characterized by three basic characteristics (programmatic, health facilities and health service providers) that should be applied. However, limited is known about the use of AFHS in the context of Nepal. This study aimed to assess the extent of AFHS utilization and associated factors among higher secondary students in the Jumla district of Nepal. A cross-sectional quantitative study was conducted in October-November 2017. Data were collected from a random sample of 528 aged 16–19 years old using a self-administered survey in their classroom. Adjusted Odds Ratios (AOR) and a 95% confidence level were estimated to measure the strength of association between the outcome variable (utilization of AFHS) and independent variable using multivariable logistic regression. Knowledge related to AFHS, measured by a seven-item scale, was based on information about the availability of AFHS. More than two-thirds (67.05%) of adolescents had utilized AFHS at least once in the last twelve months before the survey. In multivariable logistic regression analysis, knowledge level [AOR = 14.796, 95%CI (5.326–41.099)], cost of services [AOR = 2.971, 95%CI (1.764–5.003)], satisfaction from services [AOR = 1.817, 95%CI (1.037–3.185)] and availability of waiting room [AOR = 1.897, 95%CI (1.096–3.283)] were significantly associated with the utilization of AFHS. The utilization of AFHS was less than the country’s target of universal utilization in this study. Adolescents’ knowledge level about AFHS was importantly associated with its utilization. Utilization increases with lower service costs, client satisfaction, and availability of waiting rooms in the health facility. The health planners should make efforts to create a conducive environment for the adolescent by training the AFHS providers, particularly those who work in government institutions, and strengthening the awareness creation strategies among adolescents to increase the utilization of the services.

## Introduction

During adolescence (10–19 years of age), an individual faces a series of physiological, mental, and social changes, experiencing numerous changes in health status [1–4]. Around 1 in 6 persons worldwide are adolescents (1.2 billion people), and their numbers are increasing. About 80% of adolescents live in developing countries [5]. Globally, adolescents’ reproductive health needs have changed dramatically in the last 25 years, and the response to their sexual and reproductive health rights has been given immense importance. Adolescents prefer not to get married early and are more likely to use contraception to promote maternal health [6].

In South East Asian Region, undernutrition is a public health concern in the adolescent age group, and early marriage for girls is common. The total fertility rate contributed by 15–19 years old girls varies from 5%-20%. HIV prevalence among youth (15–24 years) ranges between 0.01% to 1.32%. About 95% of new HIV infections in young people in Asia are in young sex workers, young men who have sex with men, and young injecting drug users [1].

In Nepal, adolescents comprise about one-fourth of the total population [2]. In this period, physiological development does not occur completely, leading to the risk related to childbirth, causing the death of the mother and child. But nearly 3 in 10 older female adolescents (15–19 years) are already married, 17.0% had begun childbearing, 73.2% had four antenatal care (ANC) visits completed, and only 63.7% had institutional delivery [7, 8]. In 2018, the contraceptive prevalence rate (CPR) among 15–19 years old females was 7.3%, and an unmet need for family planning was 38.3% [7, 9]. The prevalence of anaemia among (15–19) years adolescent women has increased from 38.3% in 2011 to 43.6% in 2016 [7]. A recent study conducted in Kavrepalanchowk, Nepal, showed that the prevalence of anaemia among school-going adolescent girls was about 15% [10]. Nearly less than half (36%) of adolescents have adequate knowledge about menstruation, and only 13% exercise good menstrual hygiene [11, 12]. In this context, adolescents have limited access to proper information about sexual and reproductive health issues [13].

As a member of the United Nations (UN), Nepal is a part of the global initiative in which the UN has envisioned a world where every woman, child, and adolescent in every setting can enjoy their rights to physical and mental health well-being [14]. Similarly, as a signatory of the ICPD Plan of Action (1994) and different international treaties and conventions, Nepal has recognized sexual and reproductive health as a crucial aspect of overall health [15, 16]. In this context, the Government of Nepal, Ministry of Health and Population (MoHP) has developed and implemented the National Reproductive Health Strategy 1998, which clearly states adolescent sexual and reproductive health (ASRH) as one major component of reproductive health. The MoHP also devised the National Adolescent Health and Development Strategy in 2000 (revised in 2018), which includes AFHS as a key objective and comprehensive activities required to improve access to and utilization of ASRH services [17, 18]. The National Health Sector Programme III targeted implementing the National AFHS in 2000 public health institutions by 2021 [19]. It includes programmatic (involvement of adolescents in the program, males and females are equally welcomed, short waiting time, etc.), health facilities (convenient service time, convenient location, adequate space, appropriate place for registration), and health service provider (in-depth knowledge and skills, trained on ASRH issues, ensures the privacy and confidentiality, etc.) as three basic characteristics that should be applied [15]. Further, Nepal Health Sector Strategy Implementation Plan 2016–2021 has also emphasized scaling up AFHS in all local health facilities (HFs) of Nepal [19].

MoHP implemented the ASRH pilot program in 26 HFs of the Baitadi, Bardiya, Surkhet, Dailekh, and Jumla districts in 2008 [20]. As expected, the adolescent did not utilize basic health services as this provision could not address the adolescent health needs. So, the concept of upgrading HFs into AFHS was started in 2009/2010 [15]. Altogether 1355 HFs have been upgraded to AFHS across the country [18]. Similarly, AFHS centers and corners in health facilities and schools have been set up [21].

A study was conducted in the country to assess the utilization of AFHS in 2015 [17]. However, this study fails to explore factors affecting the utilization of AFHS due to the availability of limited data from the health facility. Despite being one of the first districts to implement ASRH services in the country and implementing AFHS in health facilities to increase access to adolescents, there is a paucity of AFHS service utilization and client satisfaction information in the mountainous region. This study was designed to study the prevalence and factors affecting the utilization of AFHS in the district.

Hence this study’s findings permit adolescent issues to be addressed in the context of adult professionals deciding on them. The information obtained can inform health planners to create innovative strategies that increase adolescents’ health service use.

## Methods

### Study design and study settings

Jumla is one of the districts of Karnali Province of Nepal, which is a backward district in terms of literacy rate, economic status, and other infrastructure of development. It is a remote Himalayan mountainous district of Nepal, 900 km northwest of Nepal’s capital city. In Jumla, teenage pregnancy was 22.6%, and 68% of adolescent girl has not adequate awareness about teenage pregnancy [22, 23]. Out of 30 government health facilities in the district, 15 health facilities have been upgraded to AFHS. The district was purposively selected as AFHS has been implemented in this district since 2008 [20].

A community-based cross-sectional quantitative study design was used to assess AFHS utilization and its associated factors among high school-going adolescents aged 16–19 years from October to November 2017 in the Jumla district.

### Study variables

#### Outcome variable

Utilization of AFHS was the main outcome variable and was defined as using any of the basic reproductive health (RH) services such as counseling services, voluntary counseling and testing (VCT) and HIV/AIDS services (counseling for HIV pre-test and post-test especially for prevention of mother to child transmission), family planning services, sexually transmitted infections (STIs) diagnosis and management, ANC services, safe abortion services, and other adolescent targeted general health-related services (services other than SRH services, e.g., mental illness, behavioral health problems, injuries, etc.) in the last twelve months before the survey. It was categorized as a binary outcome where; utilization of AFHS was assigned a value of 1 and non-utilization of AFHS was assigned a value of 0. The services for the last twelve months were only queried to minimize any recall bias in the study.

### Independent variables

**Fig 1:**
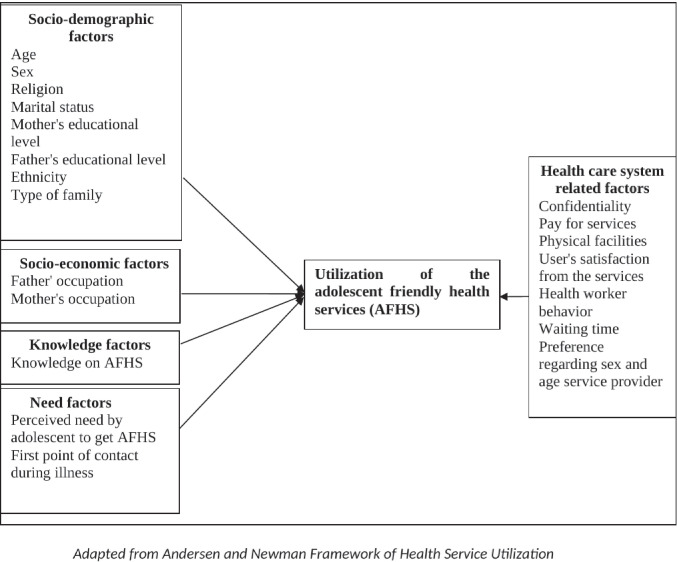
Conceptual framework of the study.

Socio-demographic characteristics such as age, sex, religion, type of family, marital status, parent’s education, parent’s occupation, knowledge about AFHS, perceived need to get AFHS, first contact point during illness, preference regarding age and sex of service providers, the welcoming behavior of service providers, confidentiality, the cost for services, physical facilities, waiting time and satisfaction from services as shown in Fig 1.

### Definition of key independent variables

***Age:*** in years, grouped into two categories, (16–17) years and (18–19) years.

***Marital status:*** grouped into two categories; Never married who had not married life time and ever married who had married at least once during life time.

***Parent’s education (father’s and mother’s education):*** Unable to read and write includes those who was unable to read and write; Able to read and write includes those who was able to read and write and also comprises formal education.

***Parent’s occupation (father’s and mother’s occupation):*** Paid occupation which has a guarantee to generate immediate money (service in government or private organization, labor and self-employment); Unpaid occupation: which has no guarantee to generate immediate money (Agriculture, Housewife, Unemployed).

***Knowledge level:*** Knowledge about AFHS was calculated based on a seven-item scale on knowledge of the availability of AFHS. Knowledge scores of 0 to 4 were categorized as low knowledge level and 5 to 7 as high knowledge level. The mean knowledge score was calculated based on the number of services reported by the respondents out of seven items.

***Perceived need to get AFHS:*** Respondents who perceived the need to get AFHS immediately after the illness develops was categorized as “As soon as the illness develops,” and who perceived the need for AFHS either after home remedies or when the condition gets worse was categorized as “not as soon as the illness develops.”

***Preference regarding the sex of service providers:*** Disregarding age, respondents who prefer same-sex service providers were categorized as health workers of the same sex and didn’t care about the sex of service providers were categorized as health workers of any sex.

***Pay for services:*** Those respondents who reported paying for the AFHS were categorized as Yes and those who reported didn’t pay for the AFHS were categorized as No.

***Satisfaction from services:*** Those respondents who reported satisfaction from the AFHS were categorized as Yes, and who didn’t report satisfaction with the AFHS were categorized as No.

***Waiting time long:*** Those respondents who reported waiting for long for AFHS were categorized as Yes, Otherwise, No.

***Physical facilities:*** Those respondents who reported that the health facility had physical facilities like a toilet facility, water supply, and waiting room were categorized as Yes, otherwise, No.

### Sampling

The sample size for the study was calculated using a single population proportion formula with the following assumptions: utilization of AFHS by the adolescent to be 34% [20], a 5% margin of error, 95% confidence level, 5% non-response rate, and a design effect of 1.5 yielded a sample size of 544 [24].

**Fig 2:**
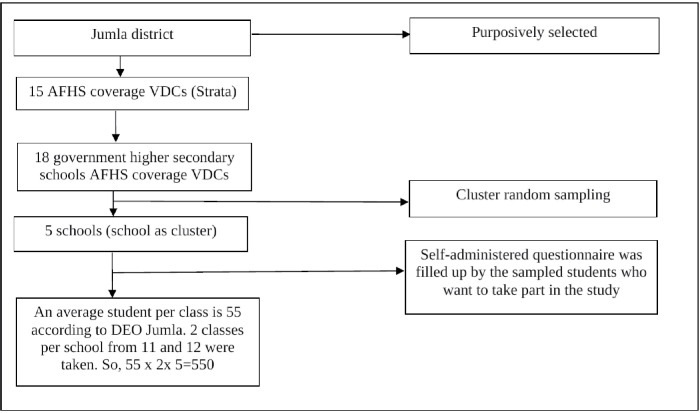
Sampling procedure.

A multistage cluster sampling procedure was used to select the study participants from all adolescents aged 16–19 years studying in grades 11 and 12 in the district. The district was stratified into 15 strata, i.e., Village Development Committee (VDC), using the district’s existing AFHS-implemented VDCs. There were altogether 18 government schools in these 15 VDCs. From 18 schools, five schools were selected as a cluster randomly for the study purpose. Out of 550 students available, 548 were approached for the study, meeting the inclusion criteria. The sampling procedure is shown in Fig 2.

### Data collection tools and techniques

The data collection tool was developed from a literature review [4, 25, 26] and different experts’ (a public health professional, an AFHS section official from MoHP, and an AFHS provider) advice. The semi-structured questionnaire in the Nepali language, which was paper-based, was pre-tested among 30 government high school students from the same district. The questionnaires were paraphrased, and sequences were aligned, maintaining its content validity after the pre-testing. Questionnaires were self-administered by the students after instructing students how to fill out the questionnaire. Students filled out the questionnaires during school hours which took about 30–45 minutes.

### Data processing and analysis

All filled questionnaires were checked for completeness, legibility, and consistency. Any incompletely filled questionnaire was excluded for data entry and analysis. The data were entered into Epi Data Entry Version 3.1. The data were exported to the statistical software package for social sciences (SPSS) Version 20 computer software for further analysis. Descriptive statistics such as frequencies, proportions, and numerical summary measures were used to describe the data. A multicollinearity test was carried out using the variance inflation factor (VIF) to see the correlation between each explanatory variable. Before inclusion in the logistic regression, the VIF of the independent variables was checked using collinearity diagnostic. There was no problem with collinearity among the independent variables, as VIF was found to be 1.02. Nagelkerke R square test and Hosmer and Lemeshow goodness of fit test was done to test whether the data fits the logistic model. The value was 0.102 indicating data fits the model. The value of the multivariate regression analysis of the study was fitted on the regression equation.

Bivariate and multivariate analyses were done to assess the association between the outcome variable and each independent variable. All the variables scored at a p-value less than or equal to 0.25 at bivariate analyses were included in multivariate analyses. The adjusted odds ratio and a 95% confidence level were estimated to identify the factors associated with AFHS utilization. The statistical significance level was measured at a p-value less than 0.05.

### Ethical considerations

The ethical approval (Ref no. 59(6-11-E)2/074/075) was obtained from the Institutional Review Committee of the Institute of Medicine, Tribhuvan University. The study permission was taken from the District Health Office and the District Education Office. The school principals, class teachers, and representatives from the school management committee were briefed on the purpose of the study.

The purpose of the study, the nature of the information needed, and the correct ways to self-administer the semi-structured questionnaire were explained to the students. The students were also asked to read and sign the first consent page on the semi-structured questionnaire. The students were assured of the confidentiality of the information and informed about their right to skip questions or withdraw anytime from the study.

The class teachers helped distribute the assent forms to each student so that their parents or guardians were informed about the purpose of the study. Students below 18 years present on the data collection day without their parents and guardians signing the assent form were excluded from the sampling frame.

## Results

Among 548 students approached for the study, 528 (96.4%) completed the survey (7 did not respond, and 13 did not complete the survey).

### Utilization of adolescent-friendly health services

More than two-thirds of adolescents reported utilizing at least one of the seven services of AFHS in the last 12 months preceding the survey. Nearly nine in ten adolescents visited for general health services, followed by safe abortion and family planning services (35.87% and 35.59%).

More than half of the study population only considered visiting AFHS after failure to solve issues themselves using home remedies such as self-care/special care such as taking a special diet, changing lifestyle, etc. Nearly one in five visited private health facilities as the first contact point for health issues.

In order to discuss problems related to sexual and reproductive health (SRH), nearly seven in ten adolescents preferred their fellow friends or peers, as shown in Table 1.

**Table 1:** Utilization of AFHS by the study population.

**Services (Multiple Answers)**	Number (%)* **n = 354**
General health services	324 (91.53)
Care of young pregnant mothers	136 (25.75)
Family Planning services	126 (35.59)
Safe Abortion Services	127 (35.87)
Counseling services	114 (32.20)
HIV counseling services	102 (28.81)
STIs diagnosis and treatment	91 (25.71)
**Perceived need to get AFHS services**	**Number (%) N = 528**
After home remedies	268 (50.75)
As soon as the illness develops	188 (35.61)
When the condition gets worse	72 (13.64)
**First contact point during the illness**	
Health Post (HP)/Primary Health Care Center (PHCC)	233 (44.13)
Private hospital	107 (20.26)
Clinics	81 (15.34)
FCHVs	64 (12.12)
Traditional healers	43 (8.14)
**Preference for communication about SRH**	
Friends/Peers	368 (69.70)
Teachers	67 (12.69)
Brother/sister	40 (7.57)
Parents	39 (7.39)
Health worker	14 (2.65)
**Preference regarding age and sex of the service provider**	
Matured and of same-sex	121 (22.92)
Young and of same-sex	116(21.97)
Matured and of any sex	106(20.07)
Young and of any sex	49 (9.28)
Doesn’t matter	136 (25.76)

*Multiple responses, General health services indicate services other than SRH services, e.g., mental illness, behavioral health problems, injuries, etc.

Similarly, 44.89% of adolescents preferred same-sex service providers, as shown in Table 1.

### Factors associated with the utilization of AFHS

The bivariate analyses showed that utilization of AFHS was similar irrespective of age group and sex of the adolescents. Adolescents who perceived the need to get AFHS as soon as the illness developed were 1.56 times more likely to utilize AFHS. Regarding physical facilities, the availability of toilets, water supply, and waiting rooms compared to no toilet, water supply, and waiting rooms increased the odds of an adolescent using AFHS with 1.84, 1.87, and 1.68, respectively.

The adolescents were more than twice likely to use AFHS if there was no service payment. Adolescents with higher knowledge levels about AFHS, compared to low knowledge about AFHS, were 15.6 times more likely to use AFHS, as depicted in Table 2.

**Table 2:** Bivariate analysis of factors associated with the utilization of AFHS N = 528.

Variables	Utilization of AFHS		Statistical values		
	Users n (%)	Non-users n (%)	Crude Odds Ratio (COR)	95% CI	p-value
**Age group**					
16–17 years	227 (66.18)	116 (33.82)	1.119	0.763–1.641	0.565
18–19 years	127 (68.65)	58 (31.35)	Ref		
**Sex**					
Male	135 (68.18)	63 (31.82)	Ref		
Female	219 (66.36)	111 (33.64)	1.086	0.746–1.582	0.667
**Marital Status**					
Never married	330 (66.13)	169 (33.87)	2.458	0.921–6.557	0.072
Ever Married	24 (82.76)	5 (17.24)	Ref		
**Mother’s education**					
Unable to read and write	308 (66.96)	152 (33.04)	Ref	0.599–1.778	0.910
Able to read and write	46 (67.65)	22 (32.35)	1.032		
**Father’s education**					
Unable to read and write	30 (75.00)	10 (25.00)	Ref		
Able to read and write	324 (66.39)	164 (33.61)	1.519	1.034–2.495	0.268
**Mother’s occupation**					
Paid	49 (68.05)	23 (31.95)	1.055	0.619–1.796	
Unpaid	305 (66.88)	151 (33.12)	Ref	0.583–2.015	0.844
**Father’s occupation**					
Paid	144 (66.05)	74 (33.95)	1.079	0.747–1.559	0.685
Unpaid	210 (67.74)	100 (32.26)	Ref		
**Knowledge level**					
High Knowledge	95 (95.96)	4 (4.04)	15.589	5.902–43.185	0.001*
Low Knowledge	259 (60.37)	170 (39.63)	Ref		
**Mean knowledge score ± S.D.**	1.12± 0.325				
**Perceived need to get AFHS**					
As soon as the illness develops	114(60.64)	74 (39.36)	1.558	1.072–2.265	0.020*
Not As soon as the illness develops	240 (70.58)	100 (29.42)	Ref		
**Preference regarding sex of service provider**					
Health workers of same-sex	171 (70.10)	73 (29.90)	1.293	0.896–1.865	0.169
Health workers of any sex	183 (64.44)	101 (35.56)			
**Pay for services**					
Yes	94 (62.25)	57 (37.75)	Ref		
No	164 (80.79)	39 (19.21)	2.550	1.578–4.120	0.001*
**Satisfaction from services**					
Yes	109 (80.15)	27 (19.85)	1.870	1.124–3.110	0.016*
No	149 (68.35)	69 (31.65)	Ref		
**Confidentiality Maintained**					
Yes	63 (78.75)	17 (21.25)	1.501	0.827–2.725	0.181
No	195 (71.17)	79 (28.83)	Ref		
**Satisfaction from the behavior of HW**					
Yes	163 (75.81)	52 (24.19)	1.452	0.903–2.333	0.124
No	95 (68.35)	44 (31.65)	Ref		
**Waiting time long**					
No	149 (77.20)	44 (22.80)	1.616	1.008–2.588	0.046*
Yes	109 (67.70)	52 (32.30)	Ref		
**Physical Facilities**					
**Toilet facility**					
Yes	231 (74.52)	79 (25.48)	1.841	0.953–3.556	0.069
No	27 (61.36)	17 (38.64)	Ref		
**Water supply**					
Yes	219 (75.26)	72 (24.74)	1.872	1.054–3.323	0.032*
No	39 (61.90)	24 (38.10)	Ref		
**Waiting Room**					
Yes	141 (77.90)	40 (22.10)	1.687	1.050–2.710	0.031*
No	117 (67.63)	56 (32.37)	Ref		

In multivariate analyses, knowledge level, pay for services, availability of waiting room, and satisfaction from services were significantly associated with utilization of AFHS, as shown in Table 3. The adolescents with higher knowledge about AFHS had odds of 14.79 times utilizing the service compared with their counterparts with lower-level knowledge about AFHS. Similarly, respondents who received free health services and reported satisfaction from services and availability of waiting rooms in the health facility were nearly three times, two times, and two times more likely to utilize AFHS, respectively, compared with their counterparts.

**Table 3:** Multivariate analysis of factors independently associated with the utilization of AFHS N = 528.

Variables	Utilization of AFHS		COR (95% CI)	p-value	AOR (95% CI)	p-value
	Users n (%)	Non- users n (%)				

**Marital Status**						
Never married	330 (66.13)	169 (33.87)	2.458 (0.921–6.557)	0.072	2.339 (0.851–6.427)	0.100
Ever Married	24 (82.76)	5 (17.24)	Ref			
**Knowledge level**						
High Knowledge	95 (95.96)	4 (4.04)	15.589 (5.902–43.185)	0.001	14.796(5.326–41.099)	<0.001*
Low Knowledge	259 (60.37)	170 (39.63)	Ref			
**Perceived need to get AFHS**						
As soon as the illness develops	114 (60.64)	74 (39.36)	1.558 (1.072–2.265)	0.020	1.391 (0.935–2.069)	0.104
Not As soon as the illness develops	240 (70.58)	100 (29.42)	Ref			
**Preference regarding sex of service provider**						
Health workers of same-sex	171 (70.10)	73 (29.90)	1.293 (0.896–1.865)	0.169	0.829 (0.560–1.228)	0.350
Health workers of any sex	183 (64.44)	101 (35.56)				
**Pay for services**						
Yes	94 (62.25)	57 (37.75)	Ref			
No	164 (80.78)	39 (19.22)	2.550 (1.578–4.120)	0.001*	2.971(1.764–5.003)	<0.001*
**Satisfaction from services**						
Yes	109 (80.15)	27 (19.85)	1.870 (1.124–3.110)	0.016*	1.817 (1.037–3.185)	0.037*
No	149 (68.35)	69 (31.65)	Ref			
**Confidentiality Maintained**						
Yes	63 (78.75)	17 (21.25)	1.501 (0.827–2.725)	0.181	1.335(0.697–2.558)	0.384
No	195 (71.17)	79 (28.83)	Ref			
**Satisfaction from the behavior of HW**						
Yes	163 (75.81)	52 (24.19)	1.452 (0.903–2.333)	0.124	1.225 (0.734–2.044)	0.437
No	95 (68.35)	44 (31.65)	Ref			
**Waiting time long**						
No	149 (77.20)	44 (22.80)	1.616 (1.008–2.588)	0.046*	0.670 (0.406–1.106)	0.117
Yes	109 (67.70)	52 (32.30)	Ref			
**Physical Facilities**						
**Toilet facility**						
Yes	231 (74.52)	79 (25.48)	1.841 (0.953–3.556)	0.069	1.174 (0.519–2.653)	0.700
No	27 (61.36)	17 (38.64)	Ref			
**Water supply**						
Yes	219 (75.26)	72 (24.74)	1.872 (1.054–3.323)	0.032*	1.276 (0.622–2.616)	0.506
No	39 (61.90)	24 (38.10)	Ref			
**Waiting Room**						
Yes	141 (77.90)	40 (22.10)	1.687 (1.050–2.710)	0.031*	1.897(1.096–3.283)	0.02*
No	117 (67.63)	56 (32.37)	Ref			

The Hosmer-Lemeshow goodness-of-fit test statistic is greater than 0.05 for all the models.

### Exposure differences for users and non-users of AFHS

The mean (±SD) age of the respondents was 17.00(±1.02) years. More than one-third of the respondents were 18–19 years old, and about three in five were females. Six in ten respondents’ fathers and one-fourth’s mothers had at least a high school education. More than half of them lived in a joint family, and 5.49% were already married, as depicted in Table 4.

**Table 4:** Exposure differences for users and non-users of AFHS (N = 528).

Variables	Users n (%)	Non-users n (%)	Total N (%)
**Age group**			
16–17 years	227 (64.12)	116 (66.67)	343 (64.96)
18–19 years	127 (35.88)	58 (33.33)	185 (35.04)
**Sex**			
Male	135 (38.14)	63 (36.21)	198 (37.50)
Female	219 (61.86)	111 (63.79)	330 (62.50)
**Class of respondent**			
Eleven	191 (53.95)	88 (50.57)	279 (51.84)
Twelve	163 (46.05)	86 (49.43)	249 (47.16)
**Ethnicity**			
Dalit	37 (10.45)	14 (8.05)	51 (9.65)
Janajati	9 (2.54)	4 (2.30)	13 (2.46)
Muslim	2 (0.56)	1 (0.57)	3 (0.57)
Brahmin/Chhetri	260 (73.45)	139 (79.89)	399 (75.57)
Others	46 (13.00)	16 (9.19)	62 (11.74)
**Religion**			
Hindu	336 (94.92)	167 (95.98)	503 (95.26)
Buddhist	11 (3.11)	3 (1.73)	14 (2.65)
Muslim	2 (0.56)	1 (0.57)	3 (0.57)
Christian	5 (1.41)	3 (1.72)	8 (1.52)
**Type of family**			
Nuclear	163 (46.04)	88 (50.57)	251 (47.54)
Joint/Extended	191 (53.96)	86 (49.43)	277 (52.46)
**Marital status**			
Ever married	330 (93.22)	169 (97.13)	29 (5.49)
Never married	24 (6.78)	5 (2.87)	499 (94.51)
**Education status of the father**			
Unable to read and write	30 (8.48)	10 (5.75)	40 (7.58)
Able to read and write	120 (33.89)	46 (26.43)	166 (31.44)
High school and above	204 (57.63)	118 (67.82)	322 (60.98)
**Educational status of the mother**			
Unable to read and write	113 (31.92)	66 (37.93)	179 (33.90)
Able to read and write	158 (44.63)	57 (32.76)	215 (40.72)
High school and above	83 (23.45)	51 (29.31)	134 (25.38)
**Occupation of father**			
Agriculture	202 (57.06)	94 (54.02)	296 (56.06)
Service	65 (18.36)	39 (22.41)	104 (19.69)
Self-employment/Business	65 (18.36)	33 (18.97)	98 (18.56)
Daily labor	16 (4.52)	3 (1.72)	19 (3.61)
Unemployed	6 (1.69)	5 (2.87)	11 (2.08)
**Occupation of mother**			
Agriculture	230 (64.97)	124 (71.26)	354 (67.04)
House wife	68 (19.21)	22 (12.64)	90 (17.04)
Service	22 (6.21)	11 (6.32)	33 (6.25)
Self-employment/Business	21 (5.93)	11 (6.32)	32 (6.10)
Unemployed	7 (1.98)	5 (2.87)	12 (2.27)
Daily Labor	6 (1.69)	1 (0.57)	7 (1.32)

There were 174 adolescents who did not use any AFHS in the past 12 months preceding this study. Nearly two in five reported a lack of awareness about AFHS, whereas one in ten complained of the lack of same-sex service providers in the health facility as a reason for the non-utilization of AFHS, as presented in Table 5.

**Table 5:** Reasons for non-utilization of AFHS n = 174.

Reasons for not utilizing AFHS among those required	Number (%)*
Lack of awareness about AFHS	67 (38.51)
Lack of time due to household work	42 (24.14)
Lack of friendly behavior of service providers	28 (16.09)
Lack of service providers of the same sex	19 (10.92)
Feeling of shyness to get the services	9 (5.17)
Lack of transportation	9 (5.17)

*Multiple responses

## Discussion

This study aimed to assess the utilization status of AFHS and factors affecting it’s utilization in the Jumla district of Nepal by providing an overview of adolescents’ ASRH service utilization, health-seeking behaviors, adolescent satisfaction, and barriers to reaching services in a remote area in the Nepalese context.

In this study, 67.05% of the adolescents utilized AFHS at least once in the last 12 months. After multivariate analysis, high knowledge level, satisfaction from services, free of cost of services, and availability of waiting rooms were positively associated with the utilization of AFHS.

About two-thirds (67.05%) of the adolescents utilized AFHS services in this study which is higher compared to other studies in Nepal, 33.80% [20] and 24.70% [27]. Since this study site was the pilot district to implement ASRH and AFHS services in 2010, the awareness could be higher in 7 years, and hence the utilization increases. Additionally, other studies were done soon (within two years of implementation), which could have affected the awareness and utilization.

The extent of using AFHS observed in this study is unsatisfactory as the ASRH program has set a target to enable all adolescents to lead a healthy and productive life by 2025 [18]. Supporting this inadequate coverage, nearly two in five adolescents (38.69%) mentioned a lack of awareness about the AFHS facilities as a significant reason for not utilizing the services. Therefore, more attention is needed from all relevant stakeholders to improve the utilization of AFHS by restraining the reasons.

In this study, 91.53% of the study participants received general health services, 25.75% got services in the care of the young pregnant mother, and 35.59% visited the AFHS center for family planning services. Sexual activity and using RH services before marriage is considered a stigma in Nepal. The use of reproductive health services among unmarried is not accepted readily in Nepalese society. However, this study shows a higher percentage (94.51%) of unmarried adolescents also utilized AFHS, as this study was conducted in high schools. This finding is similar to the results of studies done in Bhaktapur, Nepal [4, 27]. It might be due to the influence of reproductive health education in the secondary level curriculum on the utilization of AFHS, and most of them visited HFs for general health services. AFHS is a crucial source for preventing future maternal morbidity and mortality.

A similar proportion of girls and boys utilized AFHS in this study and were equally likely to utilize the service. This finding is similar to a study done in Bhaktapur, Nepal, but is, in contrast, to a study conducted in Harar, Ethiopia [4, 28]. Increasing awareness and making service providers gender-friendly can help improve the utilization of AFHS by both sexes. Besides, the sexual and reproductive health service needs are crucial to reduce adolescent female childbearing and to enhance modern CPR. There is a need to improve ways to make AFHS services friendly to females for better utilization, which is also supported by a higher (94.51%) proportion of unmarried adolescents’ utilization of our study.

Adolescents whose parents had paid occupations were more likely to use AFHS than those whose parent’s had unpaid occupations. This result contrasts the study conducted in Bhaktapur, Nepal [4]. This finding might be due to the affordability of AFHS by adolescent’s parents involved in paid occupations, as they have better economic conditions than those engaged in unpaid occupations.

About 44.13% of respondents reported visiting public HF in this study. This finding is different from the finding in Harar Town, Ethiopia, where many youths visited private institutions, but similar to Addis Ababa, Ethiopia [28]. This result might be due to the low availability of private institutions and free-of-cost services in a public health institution in Jumla. It shows that free health services should be promoted. Besides this, a significant proportion (35.60%) of adolescents went to private clinics for health services which urged making private health institutions adolescent-friendly.

One of the primary reasons for not using AFHS among adolescents was a lack of awareness about AFHS facilities, which accounts for nearly 38.51%. A study done in Nepal, in which Jumla was also one of the study areas, identified a lack of knowledge and awareness about AFHS as one of the barriers to adolescents’ utilization of health services [17]. The proportion is higher than the findings from other studies done in Ethiopia [29, 30]. This could be attributed to the extent of promoting the centers and the services in the study areas. Another possible explanation could be the difference in the age profile of the respondents included in this study and previous studies. This factor could well affect the sexual status and educational status, which might, in turn, affect the entire utilization of RH services. So, the concerned authorities and stakeholders should focus on increasing awareness about the availability of AFHS.

Knowledge level was a significant predictor of the utilization of AFHS in this study. Adolescents with high knowledge levels were more likely to utilize AFHS than adolescents with low knowledge levels. This result is similar to the study’s findings in Ethiopia (AOR = 2.340, 95% CI: 1.02–5.380) [31]. Though there is a small class interval of age, it may be due to curiosity developing around 16 and 17 years, which affects the utilization of AFHS. Knowledge is one of the predisposing factors of Andersen and Newman Framework of Health Service Utilization [32].

This study showed that the perceived need for AFHS was significantly associated with its utilization. Perceived need is one of the most immediate factors for health service use and is included as a need factor in health services utilization [32]. Those adolescents who perceived the AFHS need as soon as the illness developed were more likely to use the AFHS than those who perceived the need to get the AFHS not as soon as the illness developed. This finding is consistent with a study done in the Bhaktapur district (OR = 4.5, 95% CI: 2.01–9.94) [4]. A study conducted in India also exhibits the high demand for AFHS among adolescents [33].

Regarding health system-related factors analyzed among users for the use of AFHS. Pay for services, satisfaction from services, and availability of the waiting room were significantly associated. These factors are included as enabling factors [32]. This finding agrees with the study conducted in Malaysia in 2019 [34]. In the study done in Ethiopia, 57% of respondents stated that the service fee was expensive [29]. In a similar study done among the youth in Ethiopia, users stated long waiting times (28.40%), consultation hours were too short (25.40%), and providers were judgmental and unfriendly (23.60%) [31, 34]. In a study conducted in India, Chandigarh, a higher proportion (60.00%) of adolescents reported that they waited less than 30 minutes to receive health services in the Adolescent Friendly Health Center (AFHC), where the highest proportion (60.00%) of adolescents visited AFHC [35]. In a developing country like Nepal, where the SRH issue is stigmatized, the waiting room and the same-gender service provider influence the utilization.

This study is limited to adolescents enrolled in government schools, and out-of-school adolescents were excluded. Besides, this study has not included the duty bearers involved in managing these services, i.e., the chief of the district health office and focal person of AFHS and the service provider’s perspectives of the utilization of AFHS due to the absence of qualitative methods. These people do not directly provide health services but are involved in providing logistics, staffing, and other managerial functions. This study was determined to find utilization rather than assess the extent of using AFHS. The study’s conceptual framework guided the multivariable adjustment; however, the causal pathway method would have been a preferred method, which is one of the study’s limitations.

## Conclusions

The utilization of AFHS was less than the country’s target in this study. Adolescents with high knowledge of AFHS utilized the service more. Similarly, the low service cost, satisfaction with services, and availability of a waiting room also increased the utilization of the services. Health planners should create a conducive environment for adolescents by training AFHS providers, particularly those who work in government institutions, and strengthening the awareness creation strategies among female adolescents to increase the utilization of the services. In addition, the government should support the private sector in making private health institutions adolescent-friendly. As adolescents prefer friends/peers to communicate health problems, interventions such as peer education should be strengthened and expanded. The local government could contribute to implementing AFHS as a basic health service, as it is the responsibility of the local level as per the Constitution of Nepal 2015.

## Supporting information

S1 Questionnaire Questionnaire for data collection. (PDF)
