# Supplementary material for: Utilization of adolescent friendly health services and its associated factors among higher secondary students in mid-western Himalayan mountainous district of Nepal
Source: PLOS Glob Public Health. 2023 Mar 17;3(3):e0001616. doi: 10.1371/journal.pgph.0001616 (PMC10022795; doi:10.1371/journal.pgph.0001616)
Supplement: S1 Questionnaire — (PDF) [file pgph.0001616.s001.pdf]

Annex I: Questionnaire with informed consent in English
Informed consent form for Self-administered Questionnaire

Tribhuvan University, Institute of Medicine

Maharajgunj Medical Campus

Department of Community Medicine and Public Health

I am Mahesh Sharma, a post graduate student studying Masters in Public Health (MPH) at Maharajgunj Medical Campus, Institute of Medicine, Tribhuvan University. I am undertaking a research on "**Utilization of Adolescent friendly Health services and Associated Factors Among Higher Secondary Students in Jumla District** "and objective of the my is to find out the factors associated with utilization of Adolescent Friendly Health Services. You have to answer question regarding socio- demographic and economic conditions and information regarding the utilization of adolescent friendly health services.

Hence, I would like to request you kindly to participate in this survey and study involves no risk to you. The information given is confidential and anonymous. You would not receive direct monetary benefit for taking part in the study but the information you provide would help to increase the utilization of adolescent friendly health services. The filling of the questionnaire will take about 20-30 minutes to fill. Please feel free to ask if you have any confusion regarding questions.

Participation in this study is voluntary, and if you don't want to answer, you can leave at any time. However, I hope that you will participate in this study since your views are useful in improving adolescent friendly health services for adolescents in schools in the whole district.

Do you agree to participate? YES No

Date:.................. Signature:..............

If you agree to participate please answer the given questions.

Annex II: Self-administered Questionnaire in English

DIRECTIONS:

Do not write your name; tick only one correct response and multiple responses where applicable. Only adolescent aged between (16-19) years are eligible for this study.

SELF-ADMINISTERED QUESTIONAIRE

I. D. No.

Age (In years):

Sex:

Class:

School Name:

Part 1: Socio-demographic

Q.N.	Questions	Response	Code	Skip Pattern
1	What is your ethnicity?<br>Name of your caste	1= Dalit<br>2= Janajati<br>3= Madhesi<br>4= Muslim<br>5= Brahman/Chhetri<br>6= Others		
2	What is your religion?	1= Hindu<br>2= Buddhist<br>3= Muslim<br>4= Christian<br>5= Others (Specify....)		
3	What is the type of the family where you live?	1= Nuclear<br>2= Joint<br>3= Extended		
4	What is your current marital status?	1=Married<br>2= Divorced<br>3= Widowed<br>4= Unmarried		
5	What is the education level of your father?	1= Illiterate<br>2= Read and write only 3= Primary level (Class 1- 5)		

		4= Lower Secondary level (class 6-8)<br>5= secondary level (class 9-10)<br>6= higher secondary level (class 11-12)<br>7= Bachelor and above		
6	What is the education level of your mother?	1= Illiterate<br>2= Read and write only 3= Primary level (Class 1- 5)<br>4= Lower Secondary level (class 6-8)<br>5= secondary level (class 9-10)<br>6= higher secondary level (class 11-12)<br>7= Bachelor and above		

Part 2: Socio-economic status

**Q.N.**	**Questions**	**Response**	**Code**	**Skip Pattern**
7	What is the occupation of your father?	1= Service (government or private)<br>2= Daily labor<br>3= Self- employment/ business<br>4= Farmer<br>5= Foreign employment<br>6= Unemployed<br>7= Others (if specify)		
8	What is the occupation of your mother?	1= Housewife<br>2= service (government or private)<br>2= Self- employment/ business<br>3= Farmer<br>4= Foreign employment<br>5= Daily labor<br>6= Others (if specify)		

Part 3: Knowledge Related factors

Q.N.	Questions	Response	Code	Skip pattern
9	Have you ever seen this logo?	1= Yes<br>0= No		If No, go to Q.N.11
10	What do you think it represents?	Please write here		
11	Have you heard about Adolescent Friendly Health Services?	1= Yes<br>0= No		If No, go to Q.N. 13
12	From where did you hear about adolescent friendly health services?	1= Radio/Television<br>2= Newspaper<br>3=Poster/pamphlets<br>4=Health institution<br>5=FCHVs<br>6= Friends<br>7=Teachers<br>99= Others (specify...)		
13	What are the services provided from adolescent friendly health facility?	List the services<br>1..............<br>2..............<br>3..............		

Part 4: Health system related factors

Q.N.	Questions	Response	Code	Skip Pattern
14	When you get ill, where do you seek treatment first?	1= Dhami/Jhakris<br>2= Baidhyas<br>3= FCHVs<br>4= Pharmacy<br>5= Health Post/PHC<br>6= Private clinic<br>99= Other (Specify)...		
15	At what condition did you visit health facility for health problems?	1= As soon as the illness develops<br>2= After home remedies<br>3= As the condition becomes severe		
16	With whom do you feel comfortable to communicate the matters	1= Friends<br>2= Siblings<br>3= Parents		

	related to reproductive and sexual health?	4= Teachers<br>99= Others (Specify...)		
17	Have you used any of these services from government health facility in last 12 months?			If none of the health services has been utilized, go to Q.N.29
17.1	Counseling services	1= Yes<br>0= No		
17.2	Family Planning services	1= Yes<br>0= No		
17.3	VCT/HIV services	1= Yes<br>0= No		
17.4	Treatment of STI	1= Yes<br>0= No		
17.5	Antenatal services	1= Yes<br>0= No		
17.6	Safe abortion services	1= Yes<br>0 =No		
17.7	General health services	1= Yes<br>0= No		
18	Is the service hour feasible to you?	1= Yes<br>0= No		
19	Do you need to pay to get the health services?	1= Yes<br>0= No		
20	Are you satisfied with the services provided for sexual and reproductive health related problems?	1= Yes<br>0= No		
21	Was confidentiality maintained when service was provided?	1= Yes<br>0= No		
22	Were you provided counseling when health service was provided?	1= Yes<br>0= No		
23	Was the behavior of the health service provider friendly and non-judgmental?	1= Yes<br>0= No		
24	How long did you wait to get the services?	Please mention time.....		
25	Was the waiting period long?	1= Yes<br>0= No		
26	Are you satisfied with the physical facilities of health institution?			
26.1	Toilet	1= Yes		

		0= No		
26.2	Waiting Area	1= Yes<br>0= No		
26.3	Water supply	1= Yes<br>0= No		
27	Will you again visit the health facility in future for the health problems	1= Yes<br>0= No		If yes, go to Q.N. 30
28	Why won't you visit again?	Please list the reason here<br>1……………<br>2……………<br>3……………		
29	Why didn't you utilize any of the services listed in Q.N. 17 of health facility?	Please list here<br>1.............<br>2.............<br>3.............		
**Part 5: Health service Preferences**				
**Q.N.**	**Questions**	**Response**	**Code**	**Skip Pattern**
30	Whom do you want services related to sexual and reproductive service be provided	1= Young and of same sex<br>2= Young and of any sex<br>3= Elder and of same sex<br>4= Elder and any sex<br>5= Doesn't matter		
31	What are the services you wanted to be counseled in the adolescent friendly health services?	Please list the things to be counseled<br>1.............<br>2.............<br>3.............		
32	What are the services you wanted to be provided in the adolescent friendly health services?	Please list the things to be provided<br>1.............<br>2.............<br>3.............		

Comments and suggestions are highly welcomed

....

Annex III: Informed consent in Nepali

त्रिभुवन विश्वविधालय

चिकित्साशास्त्र अध्ययन संस्थान

महाराजगंज मेडिकल क्याम्पस

सामुदायिक चिकित्सा तथा जनस्वास्थ्य विभाग

"जुम्ला जिल्ला किशोर किशोरी मैत्री स्वास्थ्य सेवाको उपभोगमा असर पार्ने तत्वहरु" बारे अध्ययन सम्बन्ध सुसुचित स्वीकृति फराम

मेरो नाम महेश शर्मा हो । हाल म चिकित्साशास्त्र अध्ययन संस्थान महाराजगंज मेडिकल क्याम्पसमा जनस्वास्थ्य संकायको स्नाकोत्तर तह दोश्रो वर्षमा अध्ययनरत विद्यार्थी हुँ । म भक्तपुर जिल्लामा " **जुम्ला जिल्ला किशोर किशोरी मैत्री स्वास्थ्य सेवाको उपभोगमा असर पार्ने तत्वहरु**"बारे अध्ययन गर्दैछु । तपाईसंग यो विषयबारे केहि सोध्न चाहन्छु जसले गर्दा किशोर किशोरी मैत्री स्वास्थ्य सेवाको उपभोग बढ्नेछ भन्ने आशा गर्छु । तपाईलाई यी प्रश्नहरुको उत्तर दिन करिब २०-३० मिनेट आवश्यक पर्नेछ ।

तपाईले दिनु भएको जानकारीहरु यस अध्ययनको लागि मात्र प्रयोग गरिनेछ र पूर्ण रुपमा गोप्य राखिने छ । यस अध्ययनमा संलग्न हुदाँ यहाँलाई कुनै हानी नोक्सानी साथै प्रत्यक्ष फाइदा पनि हुने छैन । तर तपाईले दिनु भएका उत्तरहरुले अनुसन्धानकर्ताको उद्देश्य पूरा हुनेछ र यस अध्ययनको नतिजाले नीतिनिमताहरुलाई र कार्यक्रम निमताहरुलाई किशोर किशोरीको स्वास्थ्य अवस्था सुदृढ गर्न सहयोग पुग्ने छ ।

यस अध्ययनमा तपाईको सहभागिता स्वेच्छिक हुनेछ र अर्न्तवार्ताको क्रममा तपाईलाई कुनै पनि बेला बिचैमा अर्न्तवार्ता समाप्त गर्ने वा कुनै प्रश्नको उत्तर दिन नचहाने स्वतन्त्रता पनि हुनेछ । यदि तपाई यस अध्ययनको लागि सहभागी हुनु भई सबै प्रश्नको उत्तर दिनु भएमा म आभारी हुनेछु ।

यदि तपाईलाई मेरो अध्ययन सम्बन्धमा अन्य जिज्ञासा वा प्रश्नहरु छन् भने निर्धक्क रुपमा राख्न सक्नुहुन्छ ।

अध्ययनमा सहभागी हुन सहमत छु । ☐ अध्ययनमा सहभागी हुन सहमत छैन । ☐

सही :

मिति :

Annex IV: Self-administered Questionnaire in Nepali

उमेर (पूरा भएको वर्ष):-.....

लिङ्ग /Sex:

कक्षा:

विद्यालयको नाम:-........

खण्ड १: सामाजिक तथा जनसांख्यिक विवरण

प्र.न.	प्रश्नहरु	कोडिंग वर्गिकरण सहितका उत्तरहरु	कोड	कैफियत
१.	तपाईको थर के हो ?<br>यहाँ थर लेख्नुहोस्<br>.....................<br>यो प्रश्नमा तपाईले सहि उत्तरमा गोलो चिन्ह लगाउनु पर्दैन ।	१. दलित<br>२.जनजाति<br>३. मधेर्सा<br>४. मुस्लिम<br>५. बाह्मण/क्षेत्री<br>६. अन्य		
२.	तपाई कुन धर्म मान्नु हुन्छ ?	१. हिन्दु<br>२. बौद्ध<br>३. इस्लाम/ मुस्लिम<br>४. इसाई (क्रिश्चियन)<br>५. अन्य (खुलाउनुहोस्....)		
३.	तपाई कस्तो प्रकारको परिवारमा बस्नुहुन्छ ?	१. एकल<br>२. संयुक्त<br>३. वृहत		

४.	तपाईको हालको वैवाहिक स्थिति के हो ?	१. विवाहित<br>२. सम्बन्ध विच्छेद<br>३. विधवा/विदुर<br>४. अविवाति		
५.	तपाईको बुबाले कति पढ्नु भएको छ ?	१. असाक्षर<br>२. सामान्य लेखपढ<br>३. प्राथमिक तह (कक्षा १-५)<br>४. निम्न माध्यमिक तह(कक्षा ६-८)<br>५. माध्यमिक तह (कक्षा ९- १०)<br>६. उच्च माध्यमिक तह (कक्षा ११ - १२)<br>७. स्नातक तह वा सो भन्दा माथि		
६.	तपाईको आमाले कति पढ्नु भएको छ ?	१. असाक्षर<br>२. सामान्य लेखपढ<br>३. प्राथमिक तह (कक्षा १-५)<br>४. निम्न माध्यमिक तह(कक्षा ६-८)<br>५. माध्यमिक तह (कक्षा ९- १०)<br>६. उच्च माध्यमिक तह (कक्षा ११ - १२)<br>७. स्नातक तह वा सो भन्दा माथि		
**खण्ड २ : सामाजिक तथा आर्थिक विवरण (Socio- economic status)**				
७.	तपाईको बुबाको मुख्य पेशा के हो ?	१. जागिर वा सेवा<br>२. ब्यापार वा स्वरोजगार<br>३. कृषि		

		४. ज्याला मजदुर्रा<br>५. घर गृहर्स्था<br>६. वैदेशिक रोजगार<br>७. बेरोजगार<br>९९. अन्य(खुलाउनुहोस्......)		
८.	तपाईको आमाको मुख्य पेशा के हो ?	१. जागिर वा सेवा<br>२. ब्यापार वा स्वरोजगार<br>३. कृषि<br>४. ज्याला मजदुर्रा<br>५. घर गृहर्स्था<br>६. वैदेशिक रोजगार<br>७. बेरोजगार<br>९९. अन्य(खुलाउनुहोस्......)		

खण्ड ३ : स्वास्थ्य सेवाको ज्ञानसंग सम्बन्धित विवरण (Knowledge of health service related factors)

**प्र.न.**	**प्रश्नहरु**	**कोडिंग वर्गिकरण सहितका उत्तरहरु**	**कोड**	**कैफियत**
९.	के तपाईले यो चिन्ह देख्नुभएको छ ?	१. छ<br>०. छैन		यदि छैन भने प्र.न. ११ मा जानुहास्
१०.	यो चिन्हले के जनाउँदछ ?	कृपया यहाँ लेख्नुहोला.........		
११.	के तपाईले किशोर किशोरी मैत्री स्वास्थ्य सेवाको बारेमा सुन्नु भएको छ ?	१. छ<br>०. छैन		यदि छैन भने प्र.न. १३ मा जानुहास्

१२.	यदि सुन्नु भएको छ भने कहाँबाट सुन्नु भयो ?	१. रेडियो/ टि. भी<br>२. पत्रपत्रिका<br>३. पोस्टर/पम्फ्लेट्स<br>४. स्वास्थ्यकर्मी<br>५. महिला स्वास्थ्य स्वयं सेविका<br>६. साथी<br>८. शिक्षक/शिक्षिका<br>९९. अन्य (......)		
१३.	तपाईको विचारमा किशोर किशोरी मैत्री स्वास्थ्य सेवा प्रदान गरिने स्वास्थ्य संस्थाहरुमा के के सेवाहरु प्रदान गरिन्छन् ?	यहाँ उल्लेख गर्नुहोस् ।<br>१....................<br>२................<br>३..................<br>४................		

खण्ड ४ : स्वास्थ्य प्रणाली सम्बन्धी विवरण

१४.	तपाई यदी बिरामी हुनुभयो भने सबैभन्दा पहिले कहाँ जचाउन जानुहुन्छ ?	१. धामी/झाक्री<br>२. वैद्य<br>३. महिला स्वास्थ्य स्वयं सेविका<br>४. औषधी पसल<br>५. स्वास्थ्य चौकी/ प्राथमिक स्वास्थ्य केन्द<br>६. निजी अस्पताल<br>७. अन्य (........)		
१५.	यदि तपाईलाई कुनै यौन तथा प्रजनन् सम्बन्धी समस्याले सतायो भने कुन अवस्था पश्चात स्वास्थ्य संस्था जानुहुन्छ ?	१. बिरामी पर्ने बितिकै<br>२. घरेलु उपचार<br>३. अवस्था गम्भीर हुदै गए पछि		
१६.	तपाईलाई यौन तथा प्रजनन् स्वास्थ्य सम्बन्धी समस्याहरु कोसंग छलफल गर्न सजिलो महसुस गर्नुहुन्छ ?	१. साथी<br>२. भाइ/बहिनी<br>३. बुबा/आमा		

		४. शिक्षक/शिक्षिका<br>९९. अन्य(......)		
१७.	विगत १२ महिनामा तपाईले सरकारी स्वास्थ्य संस्थाबाट तल उल्लेखित मध्ये कुनै पनि स्वास्थ्य सेवा लिनुभएको छ ?			यदि कुनै पनि सेवा लिनु भएको छैन भने प्र.न. ३० मा जानुहोला ।
१७.१	परामर्श सेवा	१. छ<br>०. छैन		
१७.२	परिवार नियोजन सेवा	१. छ<br>०. छैन		
१७.३	एच.ई.भी/एड्स् सम्बन्धी सेवा	१. छ<br>०. छैन		
१७.४	यौन रोगहरुको उपचार सेवा	१. छ<br>०. छैन		
१७.५	गर्भवती जाँच सेवा	१. छ<br>०. छैन		
१७.६	सुरक्षित गर्भपतन सेवा	१. छ<br>०. छैन		
१७.७	सामान्य स्वास्थ्य सेवा	१. छ<br>०. छैन		
	**माथिको सेवासंग सम्बन्धित प्रश्नहरु**			
१८.	के स्वास्थ्य सेवा दिने समय तपाईलाई अनुकुल हुने समय हो ?	१. हो<br>०. होइन		
१९.	के तपाईले स्वास्थ्य सेवा लिन पैसा तिर्नु पर्छ ?	१. पर्छ<br>०. पर्दैन		
२०.	के तपाई यौन तथा प्रजनन् संग सम्बन्धित स्वास्थ्य सेवाबाट सन्तुष्ट हुनुहुन्छ ?	१. छु<br>०. छैन		
२१.	तपाईलाई सेवा प्रदान गर्दा गोपनियता सुनिश्चित गरिएको थियो ?	१. थियो<br>०. थिएन		
२२.	के माथि उल्लेखित सेवा लिँदा तपाईलाई परामर्श सेवा पनि प्रदान गरिएको थियो ?	१. थियो<br>०. थिएन		
२३.	के तपाईलाई सेवा प्रदान गर्ने स्वास्थ्यकर्मीहरुको व्यवहारप्रति सन्तुष्ट हुनुहुन्छ ?	१. थियो<br>०. थिएन		

२४.	तपाईलाई सेवा प्राप्त गर्न कति समय कुर्नु भयो ?	कृपया कुर्नु भएको समय यहाँ उल्लेख गर्नुहोस्........मिनेटस्/घण्टा		
२५.	के त्यो कुरेको समय लामो थियो ?	१. थियो<br>०. थिएन		
२६.	***के तपाई स्वास्थ्य संस्थामा भएको निम्न लिखित भौतिक सुविधाबाट सन्तुष्ट हुनुहुन्छ ?***			
२६.१	चर्पी/शौचालय	१. छ<br>०. छैन		
२६.२	प्रतिक्षालय	१. छ<br>०. छैन		
२७.३	पानी	१. छ<br>०. छैन		
२८.	के तपाई त्यो स्वास्थ्य संस्थामा भविश्यमा यौन तथा प्रजनन् सम्बन्धी सेवा लिन जानुहुन्छ ?	१. जान्छु<br>०. जान्न		यदि जानुहुन्छ भने प्र.न. मा जानुहास्
२९.	यदि माथिको स्वास्थ्य संस्थामा यौन तथा प्रजनन् सम्बन्धी सेवा लिन जानुहुन्न भने किन जानुहुन्न ?	कृपया यहाँ उल्लेख गर्नुहोस् किन जानुहुन्न<br>१.................<br>२.................<br>३.................<br>४..................		
३०.	यदि तपाईले प्र.न. १७ मा उल्लेख गरिएका सेवाहरु मध्ये कुनै पनि सेवा लिनुभएको छैन भने किन लिनु भएन ?	कृपया यहाँ उल्लेख गर्नुहोला सेवाहरु नलिनुका कारणहरु<br>१..........................<br>२.........................<br>३.........................<br>४........................		
**खण्ड ४ : स्वास्थ्य प्राथमिकता (Health Preferences)**				
३१.	तपाई यौन तथा प्रजनन् स्वास्थ्य सेवा कसले प्रदान गरेमा सेवा लिन जानुहुन्छ ?	१. युवा तथा एउटै लिगं<br>२. युवा तथा जुनसुकै लिगं<br>३. परिपक्क तथा एउटै लिगं<br>४. परिपक्क तथा जुनसुकै लिगंको		

		५. जो भए पनि केहि फरक पर्दैन		
३२.	तपाई यौन तथा प्रजनन् स्वास्थ्य सेवा दिने किशोरकिशोरी मैत्री स्वास्थ्य संस्थामा के के सेवा प्रदान गरियोस् भन्ने चाहनु हुन्छ ?			

कुनै सुझाव वा प्रतिक्रिया भए यहाँ लेख्नु होला

....
....
....
